# Efficacy and safety of berberine for dyslipidemia: study protocol for a randomized double-blind placebo-controlled trial

**DOI:** 10.1186/s13063-021-05028-8

**Published:** 2021-01-22

**Authors:** Ying Zhao, Yuan-Yuan Yang, Bao-Lin Yang, Ya-Wei Du, Da-Wei Ren, Hong-Mei Zhou, Jing Wang, Hui-Min Yang, Yao-Xian Wang, Ying-Ying Zhang, Sheng-Xian Wu

**Affiliations:** 1 Dongzhimen Hospital Affiliated to Beijing University of Chinese Medicine, Beijing, 100700 China; 2 Dongfang Hospital Affiliated to Beijing University of Chinese Medicine, Beijing, 10078 China; 3 Beijing University of Chinese Medicine, Beijing, 100029 China

**Keywords:** Berberine, Dyslipidemia, Chinese herb, Randomized controlled trial

## Abstract

**Background:**

Dyslipidemia is a major risk factor for atherosclerotic cardiovascular disease and a leading cause of death worldwide. The clinical utility of commonly used lipid-lowering drugs such as statins and fibrates is sometimes limited by the occurrence of various adverse reactions. Recently, berberine (BBR) has received increasing attention as a safer and more cost-effective option to manage dyslipidemia. Thus, a high-quality randomized controlled trial to evaluate the efficacy and safety of BBR in the treatment of dyslipidemia is deemed necessary.

**Methods/design:**

This is a randomized, double-blind, and placebo-controlled clinical trial. A total of 118 patients with dyslipidemia will be enrolled in this study and randomized into two groups at a ratio of 1:1. BBR or placebo will be taken orally for 12 weeks. The primary outcome is the percentage of low-density lipoprotein cholesterol reduction at week 12. Other outcome measures include changes in other lipid profiles, high sensitivity C-reactive protein, blood pressure, body weight, Bristol Stool Chart, traditional Chinese medicine symptom form, adipokine profiles, and metagenomics of intestinal microbiota. Safety assessment includes general physical examination, blood and urine routine test, liver and kidney function test, and adverse events.

**Discussion:**

This trial may provide high-quality evidence on the efficacy and safety of BBR for dyslipidemia. Importantly, the findings of this trial will help to identify patient and disease characteristics that may predict favorable outcomes of treatment with BBR and optimize its indication for clinical use.

**Trial registration:**

Chinese Clinical Trial Registry ChiCTR1900021361. Registered on 17 February 2019.

**Supplementary Information:**

The online version contains supplementary material available at 10.1186/s13063-021-05028-8.

## Background

Dyslipidemia is characterized by increased plasma total cholesterol (TC), low-density lipoprotein cholesterol (LDL-C), triglyceride (TG), or decreased high-density lipoprotein cholesterol (HDL-C). It is the main risk factor correlated with the occurrence and progression of atherosclerosis, cardiovascular disease (CVD), and stroke [1, 2]. As CVD remains a major cause of morbidity and mortality globally, the management of dyslipidemia is a key component of primary and secondary risk reduction strategies [3]. Statins, as the first-line therapy for dyslipidemia recommended by international clinical practice guidelines, can significantly reduce the risk of cardiovascular events [4, 5]. Nevertheless, statins are also associated with various adverse reactions such as hepatobiliary disorders, renal disease, myalgia, myositis, and rhabdomyolysis; long-term use of statins may increase the risk of incipient diabetes and cognitive dysfunction [6–11]. Other drugs such as fibrates also have similar adverse reactions, including liver toxicity, renal toxicity, and myopathy [12]. The management of dyslipidemia still remains a major challenge for patients who cannot tolerate the side effects of these classic lipid-lowering drugs (LLDs). Therefore, it is important to seek effective and safe alternatives to these LLDs.

Berberine (BBR) is an isoquinoline alkaloid isolated from various Chinese herbs, including Rhizoma coptidis (huanglian), Cortex phellodendri (huangbai), and Hydrastiscanadensis (goldenseal) [13]. It was initially used for the treatment of gastrointestinal infection. In recent years, a number of studies have suggested that BBR has a variety of pharmacological properties, such as antibacterial, antitumor, controlling arrhythmia, lowering blood lipids, lowering blood pressure, lowering blood glucose, and promoting islet cell regeneration [14–18].

The lipid-lowering effect of BBR has been supported in several studies. Kong et al. [17] reported that compared with placebo, oral BBR 0.5 g twice daily reduced the serum TC by 29%, TG by 35%, and LDL-C by 25%. Moreover, BBR combined with simvastatin showed enhanced lipid-lowering effect compared with monotherapy: the serum LDL-C was reduced by 31.8%, and reductions in TC and TG were also observed [19].

Occasionally, oral or intravenous BBR may cause adverse reactions such as nausea, vomiting, constipation, hypertension, respiratory failure, and paresthesias. Rare adverse effects including headache, skin irritation, facial flushing, headache, and bradycardia have also been reported [20]. The therapeutic dose of BBR (0.5 g) is generally safe and well-tolerated, and gastrointestinal reactions are most often caused by overdose (≥ 4 g).

A recent systematic review and meta-analysis demonstrated that BBR could significantly reduce the levels of TC, LDL-C, and TG in patients with dyslipidemia [13]. Unfortunately, these studies were limited by their high heterogeneity and high risk of biases. Kong et al. [17] proved that BBR treatment significantly improved lipid profiles; however, the randomization, blinding procedure, sample size calculation, and detailed inclusion and exclusion criteria have not been mentioned, and a lack of restriction on the other medications could have influenced the results. As there has been a lack of large-scale, high-quality randomized controlled trials on this possibility at the population level, this randomized, double-blind, placebo-controlled clinical trial was designed to determine the lipid-lowering efficacy and safety of BBR in treating dyslipidemia.

## Methods/design

### Trial objective

The objectives of this trial are as follows:

1. To assess the efficacy and safety of BBR for dyslipidemia
2. To identify patient and disease characteristics that may predict favorable outcomes of treatment with BBR and optimize its indication for clinical use

### Design

**Fig. 1:**
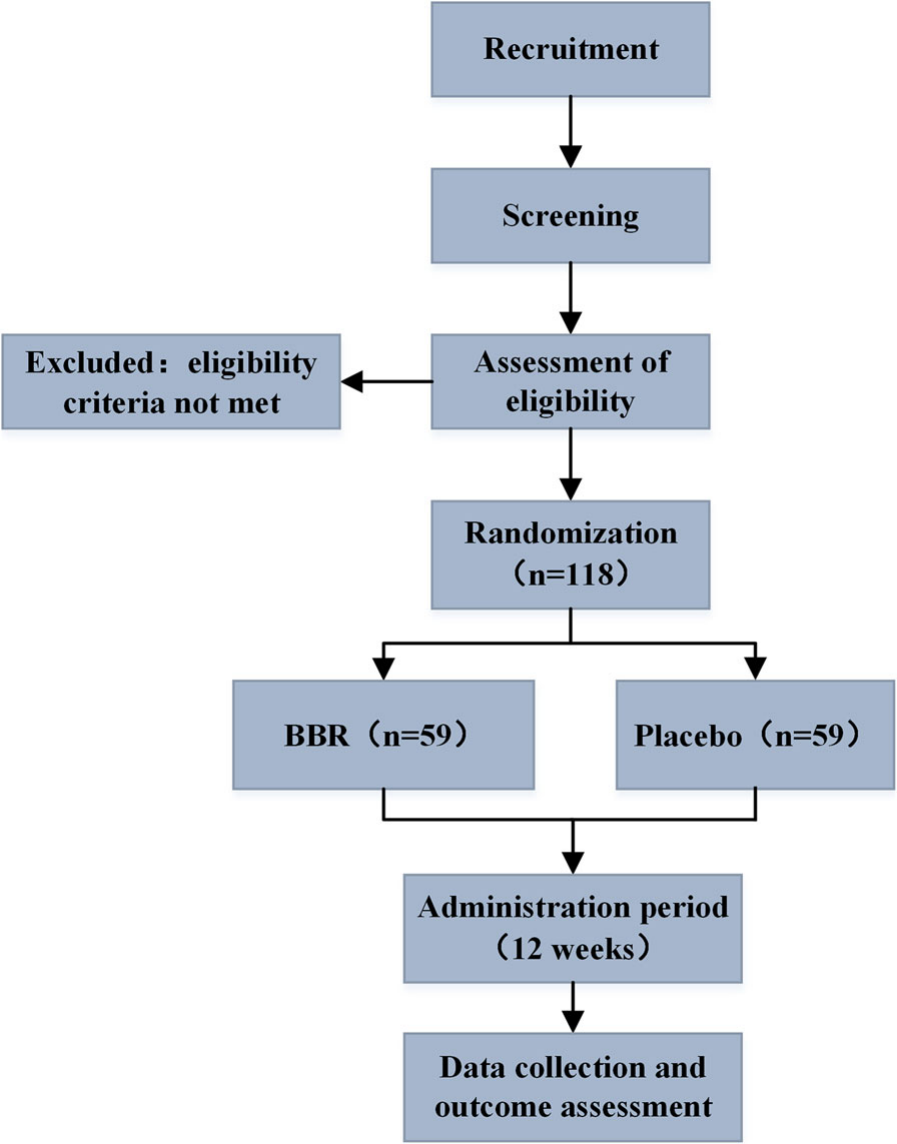
Study flowchart. BBR, berberine

This is a study protocol for a randomized, double-blind, placebo-controlled clinical trial with a treatment period of 12 weeks, which will be conducted between April 2019 and December 2020. Eligible patients will be randomized at a ratio of 1:1 into either the BBR group or the placebo group. The protocol was developed according to Consolidated Standards of Reporting Trials (CONSORT) statement [21], Standard Protocol Items: Recommendations for Interventional Trials (SPIRIT) 2013 [22], and SPIRIT 2013 explanation and elaboration: guidance for protocols of clinical trials [23]. A total of 4 visits will be scheduled at baseline (visit 1) and weeks 4 (visit 2), 8 (visit 3), and 12 (visit 4) to assess treatment efficacy, safety, and adverse events. This 12-week treatment period was set based on our clinical experience and previous pilot studies [13, 17]. Additionally, potential predictive factors involving patient and disease characteristics have been collected in a comprehensive manner. Figure 1 shows the flow diagram of the trial.

The protocol design is based on the Standard Protocol Items: Recommendations for Interventional Trials (SPIRIT) Checklist (Additional file 1).

### Institutional setting

This study will be conducted in Dongzhimen Hospital Affiliated to Beijing University of Chinese Medicine (DHABUCM), Beijing, China.

### Recruitment and participants

#### Recruitment procedures

All participants will be recruited in DHABUCM. Advertisements and colored fliers will also be placed inside the hospital. The contents of the recruitment advertisement include a brief introduction to dyslipidemia and BBR, the inclusion criteria, the intervention, and the contact information of the investigators. Those who are willing to participate in this trial may contact the research assistants by telephone. Once they have volunteered to participate, they will be assessed according to the inclusion and exclusion criteria. Eligible patients will be asked to sign the written informed consent form before the initiation of any study-related procedures. The detailed information about the participants will never be revealed to any individual or organization relevant to the study.

### Research population and inclusion criteria

#### Diagnostic criteria

The diagnosis of dyslipidemia is made according to the Guidelines for the Prevention and Treatment of Dyslipidemia in Chinese Adults (revised in 2016) [24]: TC ≥ 6.2 mmol/L or LDL-C ≥ 4.1 mmol/L or HDL-C < 1.0 mmol/L or TG ≥ 2.3 mmol/L, occurring in the absence of any secondary causes.

#### Inclusion criteria

1. Diagnosis of primary dyslipidemia
2. LDL-C ≥ 4.1 mmol/L and/or TG ≥ 2.3 mmol/L
3. Age between 18 and 70 years, male or female
4. Having not taken any LLDs within the past 1 month
5. Able to adhere to long-term treatment
6. Having voluntarily given their informed consent

#### Exclusion criteria

1. History of myocardial infarction, stenting, bypass surgery, or stroke within the past 4 weeks
2. Combined with diabetes, nephrotic syndrome, or renal insufficiency
3. Combined with poorly controlled hypothyroidism or other endocrine diseases
4. Alanine aminotransferase (ALT) and/or aspartate aminotransferase (AST) ≥ 2 times the upper limit of normal
5. Combined with severe or active liver diseases
6. Combined with severe or any other life-threatening disease
7. Allergy to components of the product or allergic constitution
8. Combined with a mental disorder
9. Participated in clinical trials of other drugs in the past 4 weeks

#### Randomization

The stratified blocked randomization method will be used. The statistical software SAS 9.2 (SAS Institute Inc., Cary, USA) will be used by an independent statistician who is not involved in the implementation or statistical analysis of the trial. A random code list will be generated, and eligible participants will be randomly assigned to either the BBR group or the placebo group at a ratio of 1:1.

#### Blinding and unblinding procedures

A two-level blinding design will be used, with the first level being by group (group A and group B) according to case number and the second level by treatment (BBR and placebo).

The random code list will be created by a statistical agency, the two-level treatment codes will be sealed separately in duplicate, with a copy being kept by DHABUCM and the National Drug Clinical Trial Institution. Treatment allocations will be blinded to the participants, clinicians, research assistants, drug managers, statisticians, and other staff members and will not be revealed until the study is completed. The study would have been considered invalid if any treatment code disclosure arose from any non-prescribed circumstances and if it affected the objectivity of the data obtained from the study.

Whether the drug packaging meets the requirements of a double-blind clinical trial will be checked, and the drug quality inspection report will be created. The entire drug coding process is documented by the blind editor, as part of the documentation for the clinical trial. Each coded test drug will have a corresponding emergency letter, describing the subject’s medication, treatment methods, organization, and address to which requests for emergency unblinding should be immediately reported. The coded drugs will be sent to the study center together with the corresponding emergency letters.

In case of emergency (such as serious adverse reactions), or when the patient needs to be rescued and must know what kind of treatment the patient has received, the corresponding emergency letters shall be opened with the presence of the investigators and the administrator of research organization. Once the emergency letter is opened, the numbered subject will withdraw from the study. The investigators will record the reason in the case report form (CRF). All emergency letters will be collected with the CRFs for blind review at the end of the trial.

### Interventions

#### Lifestyle interventions

The participants in both groups will receive health education about diet and exercise according to the Guidelines for the Prevention and Treatment of Dyslipidemia in Chinese Adults (revised in 2016) [24]. Regulation of diet and exercise of the subjects will be regarded as very important in this study. A standardized meal suggestion (30 kcal/kg ideal weight/day) will be provided, which includes a reasonable match of calories, protein and carbohydrates, low-cholesterol, low-salt, and low-sugar diet. Individuals will be recommended to do moderate-intensity metabolic exercise for 30 min, 5–7 days a week. Participants will be asked to be strict about their diet and to avoid short-term or high-intensity exercise. Risk factors, such as smoking and alcoholism, will be controlled strictly. Investigators will emphasize the importance of diet and exercise interventions at each treatment visit.

#### Pharmacologic treatments

A total of 118 patients with primary dyslipidemia will undergo baseline data acquisition and lifestyle guidance and be randomly assigned into two groups: BBR (0.5 g, twice daily) or placebo. BBR or placebo will be administrated orally for 12 weeks. BBR and placebo were produced by the same pharmaceutical company (Shanxi Zhendong Pharmaceutical Co., Ltd. Lot No.:20190301). They have the same appearance, size, odor, and taste.

During each visit, the observer needs to record in detail whether the patient takes the medicine on time, and withdraws the residual drugs, so as to assess the patient’s medication compliance: drug compliance = actual total amount of medicine taken/total amount of medicine required by the protocol × 100%. Any drug administered during the study must be recorded and described in detail in CRFs, including the specific type of drug (trade name or generic name), the indications for use, the date, and the dose. Any lipid-lowing therapy except for the experimental drugs will be banned or restricted during the study.

### Outcomes

#### Primary outcome

The primary outcome is the percentage change in LDL-C from baseline at week 12.

#### Secondary outcomes

The following are the secondary outcomes:

1. Lipid profiles: TC, TG, HDL-C, and LDL-C will be measured at baseline (visit 1) and weeks 4 (visit 2), 8 (visit 3), and 12 (visit 4).
2. Hs-CRP, BP, and body weight will be measured at baseline (visit 1) and weeks 4 (visit 2), 8 (visit 3), and 12 (visit 4).
3. BSC: the participants will be asked “which type of stool they have” at each visit during the treatment period.
4. TCM symptom form (according to the Clinical Research Guidance of New Investigational Drug in TCM [25]) will be evaluated at baseline (visit 1) and weeks 4 (visit 2), 8 (visit 3), and 12 (visit 4).
5. Adipokine profiles [fibroblast growth factor 21 (FGF21), adiponectin (APN), hormone-sensitive lipase (HSL)] will be assessed based on the blood specimens collected at baseline (visit 1) and week 12 (visit 4).
6. Intestinal microbiota will be analyzed by the metagenomics based on the stool specimens collected at baseline (visit 1) and week 12 (visit 4).

#### Safety assessments

The following are the safety assessments:

1. General physical examination
2. Blood and urine routine tests, ALT, AST, BUN, and Cr
3. Adverse events

#### Adverse event monitoring

When a serious adverse event happens, investigators must fill out the “SAE form” and report it to the major investigator and center ethics committee immediately. The event should be reported to the safety supervision division of the State Food and Drug Administration within 24 h.

The major investigator must offer corresponding treatment according to the drugs and symptoms, then record and sign at the CRFs in detail. The inspector must be informed of the treating processes.

All adverse events should be recorded in time and tracked until properly resolved or stabilized. Abnormal indexes in physical and chemical examinations should be checked until normal.

#### Participant timeline

The timeline for participant registration, intervention, and assessment is shown in Table 1, based on the Standard Protocol Items: Recommendations for Interventional Trials (SPIRIT) guideline (Additional file 1).

**Table 1:** Content of data capture

Research stage	Baseline	Treatment period		
Item	0	4 weeks ± 3 days	8 weeks ± 3 days	12 weeks ± 3 days
Visiting no.	V1	V2	V3	V4
**Demographic data/baseline**				
Inclusion/exclusion criteria	√			
Informed consent	√			
History of disease and treatment	√			
Physical examination	√	√	√	√
Complications and concomitant medications	√			
**Outcome assessments**				
Lipid profiles	√	√	√	√
Hs-CRP	√	√	√	√
Blood pressure	√	√	√	√
Body weight	√	√	√	√
Bristol Stool Chart	√	√	√	√
TCM symptom form	√	√	√	√
Adipokine profiles	√			√
Intestinal microbiota	√			√
**Safety assessment**				
Blood routine measurements	√			√
Urine routine measurements	√			√
Liver function (ALT, AST)	√			√
Kidney function (BUN, Cr)	√			√
Adverse events	√	√	√	√
**Others**				
Randomization	√			
Drug distribution	√	√	√	
Drug recycling and counting		√	√	√
Record concomitant medications		√	√	√
Study conclusion				√

√: item need to be done during different period

#### Data collection

Laboratory parameters will be used for validity and safety assessments (see Table 1 for details). At the beginning of the clinical study, a detailed description about the biological specimen collection manual will be provided to researchers. Lipid profiles, hs-CRP, blood and urine routine, ALT, AST, BUN, and Cr will be measured by the laboratory department. For adipokine profile assay (FGF21, APN, HSL), 5 ml whole blood will be taken and kept at room temperature for 0.5 h. Centrifugation will be performed by 3000 r min^−1^ at 4 °C for 10 min, and the separated serum will be collected into cryopreservation tubes for storage at − 80 °C. Stool specimens in the size of soybeans will be placed into dry, sterile collection tubes and stored at − 80 °C for cryopreservation. Biological specimens collected in the study will be stored in the biological sample bank of DHABUCM.

#### Data management and quality assurance

Before the conduction of the clinical trial, all investigators and relevant staff including clinicians, research assistants, and drug managers will be uniformly trained regarding the trial-specific process. Investigators will fill in the CRFs according to the subjects’ hospitalization records and original observation records. All collected data will be checked regularly by the inspectors so as to avoid mistakes and omissions. Original record of any modification shall be clearly visible, and the corrections shall be signed and dated by the investigator. And then, the CRFs shall be submitted to the clinical trial data administrators. This transfer process between investigators, inspectors, and data administrators needs to be recorded and saved. Data will be checked again by the data administrator before data entry. Any questions and answers will be kept as query tables for future reference.

Double data entry of CRFs will be conducted independently. The data manager should check the database with the major investigator for data scope and logical content. After data entry and verification as required, the original CRFs shall be archived and saved in order, and filled with the search catalog. Electronic data files including databases, inspection procedures, analysis procedures, analysis results, codebooks, and description files, should be stored in categories, and multiple backups should be saved properly on different record media. All the original files shall be kept within the prescribed period according to China’s Drug Clinical Trial Quality Management Standard.

#### Sample size calculation

The sample size for this trial was calculated based on the primary outcome using the PASS 11.0 (NCSS LLC, Kaysville, UT, USA) software. According to the previous results of berberine on dyslipidemia [17, 26], the percentage reduction in LDL-C (the primary outcome) is 25% in the berberine group and 5% in the placebo group. Forty-seven patients in each group can achieve 80% power and rule out type I error of 5% by using a two-sided test. We expect that 20% of the participants may be lost to follow-up. Therefore, the sample size in each group should be adjusted to 59. In total, 118 participants will be enrolled in this trial.

#### Statistical analysis

Statistical analysis of all data will be performed by a dedicated statistician in a blinded manner. All statistical analyses of the data will be performed using the SAS 6.12 (SAS Institute Inc., Cary, NC, USA) software.

The analysis data set will consist of a full analysis set (FAS), a per-protocol set (PPS), and a safety set (SS). FAS: according to the principle of analysis for intention to treat (ITT), participants will be rejected by the smallest and reasonable method. The last observation carried forward (LOCF) method will be used for the missing data supplement. The PP data set will include only participants who adhered to the study protocol and completed the clinical study. The minimum compliance rate for participants taking the investigational drugs in the PP data set is 80%. The SS will include information on safety records, including adverse events and laboratory test results. Quantitative indices recorded will include the numbers of cases, mean ± SD, median, and maximum and minimum values; qualitative or grade indices will be recorded using frequency and percentage. Two-sided tests will be used in all cases, and *P* ≤ 0.05 will be considered to be statistically significant.

From baseline to each time point, discrete variables will be described with frequencies and percentages, and continuous variables will be described with either mean and standard deviation for data with normal distribution or median and interquartile range for non-normally distributed data. For comparisons between the experimental and control groups at each time point, a chi-square test or Fisher’s exact test will be used for discrete variables, and Student’s *t* test or the Wilcoxon-Mann-Whitney *U* test will be used for continuous variables.

The analysis of concomitant medications will be analyzed by the chi-square test or Fisher’s exact test. The analysis of treatment compliance should include both the experimental drug and the basic treatment, and the chi-square test/Fisher exact probability method will be used for comparison.

All adverse events during the trial should be described in the form with the type, severity, and relationship with the experimental drug. Adverse events will be classified into all adverse events and related adverse events, among which, for conservative consideration, will include “definitely related,” “probably related,” “possibly related,” and “suspected related” to the drug.

#### Protocol amendments

A formal amendment to the protocol will be required and must be approved by the major investigator and the Ethics Committee of the DHABUCM prior to implementation, if modifications may affect the conduct of the study, patients’ potential benefit or safety.

#### Monitoring and inspection

Data administrators should conduct regular monitoring to ensure that the data are adequately recorded in the case report forms and recorded prompt, accurate, and complete. The project management group will carry on conventional and emphasis inspection on every step of the research in order to evaluate whether the process of study is in accord with the protocol.

## Discussion

Several clinical studies of the efficacy of BBR in treating dyslipidemia have been published. A recent systematic review reported that the methodological quality of the majority of these trials was generally low in terms of random sequence generation, allocation concealment, blinding, and incomplete outcome data. Thus, selection bias, performance bias, detection bias, attrition bias, and confounding bias might exist [13]. Therefore, the evidence for the use of BBR in patients with dyslipidemia is not conclusive, and more rigorously designed studies with larger sample sizes are required. This is the study protocol of a randomized, double-blinded, placebo-controlled trial which aims to evaluate the efficacy and safety of BBR for dyslipidemia compared with placebo after 12 weeks of treatment. Our study will recruit a total of 118 participants to compare the effect of BBR with that of placebo in patients with dyslipidemia. The methods used for randomization, treatment concealment, and blinding are at low risk of bias and will be described in detail in the final report. The dose of BBR is 500 mg twice daily, within the range of active daily doses recommended by the consensus of the international lipid expert panel [27]. Furthermore, blood and stool samples will be kept to explore the potential lipid-lowering mechanism of BBR.

Meta-analysis showed that BBR could significantly reduce the levels of TC, LDL-C, and TG and also increase the level of HDL-C when used alone [27]. The lipid-lowering effect of BBR may be achieved through multiple mechanisms, including upregulating the expression of LDL-receptor (LDLR) [17], downregulating the expression of fat synthesis gene through activate adenosine monophosphate-activated protein kinase (AMPK) [28], downregulating the PPAR gamma gene [29], and increasing the activity of lipoprotein lipase (LPL) [30]. However, the main target of its lipid-lowering effect still needs further investigation.

### Trial status

This trial is currently recruiting patients. The recruitment process will be conducted between April 2019 and December 2020.

## Supplementary Information

**Additional file 1.** Standard Protocol Items: Recommendations for Interventional Trial (SPIRIT) 2013 Checklist: recommended items to address in a clinical trial protocol and related documents.

## Data Availability

All the individual participant data (text, tables, figures, and appendices) collected during the trial after deidentification will be available for anyone who wishes to access the data immediately following publication in accordance with the FAIR principles.

## Abbreviations

ALT: Alanine transaminase
AMPK: AMP-dependent protein kinase
AST: Aspartate aminotransferase
BBR: Berberine
BP: Blood pressure
BUN: Urea nitrogen
BSC: Bristol Stool Chart
CRF: Case report form
Cr: Creatinine
DHABUCM: Dongzhimen Hospital Affiliated to Beijing University of Chinese Medicine
FAS: Full analysis set
HDL-C: High-density lipoprotein cholesterol
hs-CRP: High-sensitivity C-reactive protein
LDL-C: Low-density lipoprotein cholesterol
LDLR: LDL receptor
LLDs: Lipid-lowering drugs
LPL: Lipoprotein lipase
PPS: Per-protocol set
SS: Safety set
TC: Total cholesterol
TCM: Traditional Chinese medicine
TG: Triglyceride
